# Supplementary material for: Pre-adult aggression and its long-term behavioural consequences in crickets
Source: PLoS One. 2020 Mar 26;15(3):e0230743. doi: 10.1371/journal.pone.0230743 (PMC7098602; doi:10.1371/journal.pone.0230743)

PLOS ONE

Pre-adult aggression and its long-term behavioural consequences in crickets

--Manuscript Draft--

**Manuscript Number:**	PONE-D-19-35154
**Article Type:**	Research Article
**Full Title:**	Pre-adult aggression and its long-term behavioural consequences in crickets
**Short Title:**	pre-adult aggression in crickets
**Corresponding Author:**	Paul Anthony Stevenson<br>Universität Leipzig<br>GERMANY
**Keywords:**	agonistic behaviour; development; animal personality; experience dependent plasticity; insects; loser effect; winner effect; social defeat stress; octopamine; adult specific behaviour
**Abstract:**	Social experience, particularly aggression, is considered a major determinant of consistent inter-individual behavioural differences between animals of the same species and sex ("animal personality"). We investigated the influence of pre-adult aggressive experience on future behaviour in male, last instar nymphs of the cricket Gryllus bimaculatus. We found that aggressive interactions between male nymphs are far less fierce than for adults in terms of duration and escalation. This appears to reflect immaturity of the sensory apparatus for releasing aggression, rather than the motor system controlling it. First, a comparison of the behavioural responses of nymphs and adults to mechanical antennal stimulation using unwashed and washed antennae taken from nymphs and adults, indicate that nymphs neither respond to nor produce sex-specific cuticular semiochemicals important for releasing aggressive behaviour in adults. Second, treatment with the octopamine agonist chlordimeform could at least partially compensate for this deficit. In further contrast to adults, which become hyper-aggressive after victory, but submissive after defeat, winner and loser effects are not apparent in nymphs. Aggressive competition between nymphs thus appears to have no consequence for future behaviour in crickets. However, male nymphs were often seen to be attacked by adult males, but not by adult females. Furthermore, observations of nymphs raised in the presence, or absence of adult males, revealed that social subjugation by adult males leads to reduced aggressiveness and depressed exploratory behaviour when the nymphs become adult. We conclude that social subjugation by adults during pre-adult development of nymphs is a major determinant of inter-individual behavioural differences in adult crickets.
**Order of Authors:**	Julia Sophie Balsam
	Paul Anthony Stevenson
**Additional Information:**	
**Question**	**Response**
**Financial Disclosure**<br><br>Enter a financial disclosure statement that describes the sources of funding for the work included in this submission. Review the submission guidelines for detailed requirements. View published research articles from *PLOS ONE* for specific examples.<br><br>This statement is required for submission and **will appear in the published article** if the submission is accepted. Please make	This study was financed by a grant of the Deutsche Forschungsgemeinschaft (https://www.dfg.de/en/) to PAS, Grant Nr. STE 714/5-1. The funders had no role in study design, data collection and analysis, decision to publish, or preparation of the manuscript.

Powered by Editorial Manager® and ProduXion Manager® from Aries Systems Corporation

sure it is accurate.<br><br>**Unfunded studies**<br>Enter: *The author(s) received no specific funding for this work.*<br><br>**Funded studies**<br>Enter a statement with the following details:<br>• Initials of the authors who received each award<br>• Grant numbers awarded to each author<br>• The full name of each funder<br>• URL of each funder website<br>• Did the sponsors or funders play any role in the study design, data collection and analysis, decision to publish, or preparation of the manuscript?<br>• **NO** - Include this sentence at the end of your statement: *The funders had no role in study design, data collection and analysis, decision to publish, or preparation of the manuscript.*<br>• **YES** - Specify the role(s) played.<br><br>* typeset	
**Competing Interests**<br><br>Use the instructions below to enter a competing interest statement for this submission. On behalf of all authors, disclose any competing interests that could be perceived to bias this work—acknowledging all financial support and any other relevant financial or non-financial competing interests.<br><br>This statement **will appear in the published article** if the submission is accepted. Please make sure it is accurate. View published research articles from *PLOS ONE* for specific examples.	The authors have declared that no competing interests exist.

Powered by Editorial Manager® and ProduXion Manager® from Aries Systems Corporation

**NO authors have competing interests**<br><br>Enter: *The authors have declared that no competing interests exist.*<br><br>**Authors with competing interests**<br><br>Enter competing interest details beginning with this statement:<br><br>*I have read the journal's policy and the authors of this manuscript have the following competing interests: [insert competing interests here]*<br><br>* typeset	
**Ethics Statement**<br><br>Enter an ethics statement for this submission. This statement is required if the study involved:<br><br>• Human participants<br>• Human specimens or tissue<br>• Vertebrate animals or cephalopods<br>• Vertebrate embryos or tissues<br>• Field research<br><br>Write "N/A" if the submission does not require an ethics statement.<br><br>General guidance is provided below. Consult the submission guidelines for detailed instructions. **Make sure that all information entered here is included in the Methods section of the manuscript.**	N/A - Study on invertebrates

Powered by Editorial Manager® and ProduXion Manager® from Aries Systems Corporation

**Format for specific study types**<br><br>**Human Subject Research (involving human participants and/or tissue)**<br>• Give the name of the institutional review board or ethics committee that approved the study<br>• Include the approval number and/or a statement indicating approval of this research<br>• Indicate the form of consent obtained (written/oral) or the reason that consent was not obtained (e.g. the data were analyzed anonymously)<br><br>**Animal Research (involving vertebrate animals, embryos or tissues)**<br>• Provide the name of the Institutional Animal Care and Use Committee (IACUC) or other relevant ethics board that reviewed the study protocol, and indicate whether they approved this research or granted a formal waiver of ethical approval<br>• Include an approval number if one was obtained<br>• If the study involved *non-human primates*, add *additional details* about animal welfare and steps taken to ameliorate suffering<br>• If anesthesia, euthanasia, or any kind of animal sacrifice is part of the study, include briefly which substances and/or methods were applied<br><br>**Field Research**<br><br>Include the following details if this study involves the collection of plant, animal, or other materials from a natural setting:<br>• Field permit number<br>• Name of the institution or relevant body that granted permission	
**Data Availability**<br><br>Authors are required to make all data underlying the findings described fully available, without restriction, and from the time of publication. PLOS allows rare exceptions to address legal and ethical concerns. See the PLOS Data Policy and FAQ for detailed information.	Yes - all data are fully available without restriction

A Data Availability Statement describing where the data can be found is required at submission. Your answers to this question constitute the Data Availability Statement and **will be published in the article**, if accepted.<br><br>**Important:** Stating 'data available on request from the author' is not sufficient. If your data are only available upon request, select 'No' for the first question and explain your exceptional situation in the text box.<br><br>Do the authors confirm that all data underlying the findings described in their manuscript are fully available without restriction?	
**Describe where the data may be found in full sentences. If you are copying our sample text, replace any instances of XXX with the appropriate details.**<br><br>• If the data are **held or will be held in a public repository**, include URLs, accession numbers or DOIs. If this information will only be available after acceptance, indicate this by ticking the box below. For example: *All XXX files are available from the XXX database (accession number(s) XXX, XXX.*).<br>• If the data are all contained **within the manuscript and/or Supporting Information files**, enter the following: *All relevant data are within the manuscript and its Supporting Information files.*<br>• If neither of these applies but you are able to provide **details of access elsewhere**, with or without limitations, please do so. For example:<br><br>*Data cannot be shared publicly because of [XXX]. Data are available from the XXX Institutional Data Access / Ethics Committee (contact via XXX) for researchers who meet the criteria for access to confidential data.*<br><br>*The data underlying the results presented in the study are available from (include the name of the third party*	All relevant data are within the manuscript and its Supporting Information files.

and contact information or URL).<br>• This text is appropriate if the data are owned by a third party and authors do not have permission to share the data.<br><br>* typeset	
Additional data availability information:	

Manuscript.docX Click here to download Manuscript Manuscript preadult aggression.docx

Pre-adult aggression and its long-term behavioural consequences in crickets

Julia S. Balsam and Paul A. Stevenson*
Institute of Biology, Leipzig University, Leipzig, Germany

*** Correspondence:**
Prof. Dr. Paul A. Stevenson
Faculty of Life Sciences
Institute of Biology
Leipzig University
Talstr. 33
Leipzig, D-04103, Germany
stevenson@rz.uni-leipzig.de
Tel.: ++(49) (0) 341 973 6879

Abstract

avoid here, discuss later

Social experience, particularly aggression, is considered a major determinant of consistent inter-individual behavioural differences between animals of the same species and sex (“animal personality”). We investigated the influence of pre-adult aggressive experience on future behaviour in male, last instar nymphs of the cricket *Gryllus bimaculatus*. We found that aggressive interactions between male nymphs are far less fierce than for adults in terms of duration and escalation. This appears to reflect immaturity of the sensory apparatus for releasing aggression, rather than the motor system controlling it. First, a comparison of the behavioural responses of nymphs a[illegible] adults to mechanical antennal stimulation using unwashed and washed antennae t[illegible] from nymphs and adults, indicate that nymphs neither respond to nor produce sex-specific cuticular semiochemicals important for releasing aggressive behaviour in adults. Second, treatment with the octopamine agonist chlordimeform could at least partially compensate for this deficit. In further contrast to adults, which become hyper-aggressive after victory, but submissive after defeat, winner and loser effects are not apparent in nymphs. Aggressive competition between nymphs thus appears to have no consequence for future behaviour in crickets. However, male nymphs were often seen to be attacked by adult males, but not by adult females. Furthermore, observations of nymphs raised in the presence, or absence of adult males, revealed that social subjugation by adult males leads to reduced aggressiveness and depressed exploratory behaviour when the nymphs become adult. We conclude that social subjugation by adults during pre-adult development of nymphs is a major determinant of inter-individual behavioural differences in adult crickets.

normal

may be treated? washed sounds a bit strange or may be *hexane-washed*

Introduction

needs a better definition, not just a note

Experiencing aggression, particularly social subjugation (social defeat stre[illegible] recognised as a major factor inducing depression and depression-like symptoms in humans [1] and consistent inter-individual behavioural differences ("animal personality") in vertebrates and invertebrates alike [2]. In this paper, we investigate the influence of pre-adult agonistic experience in male, last instar nymphs of the field cricket, *Gryllus bimaculatus*, which has advanced to a model system for studying the neurochemical control of aggression [3]. Pre-adult exposure to aggression is associated with various long-term, detrimental effects on behaviour in mammals including man [4,5] together with an increased risk for mental health problems [6]. However, although agonistic behaviour in pre-adult invertebrates is widely reported, we know of no definitive research describing its consequence for their future behaviour, in particular when adult. Nonetheless, male cricket nymphs are reported to exhibit "distinctive aggressive behaviours" that are "similar to those adopted by adults during aggressive disputes", whereby individuals that were "successful in competition monopolised access to food resources" [7]. This source also reports that "stress induced by increases in larval competition caused reductions in realised adult body size and decreased survival". The population density of nymphal crickets, which might also be expected to increase competition, was not, however, found to influence adult aggression or exploratory behaviour ("boldness", [8,9]). From studies to date it remains unclear, whether competition between nymphs establishes dominant-subordinate relationships as in adults, with potentially long lasting consequences for future behaviour. As in most animals [10], adult male crickets that win an agonistic encounter are subsequently more aggressive [11], whereas losers become submissive [12-14]. Moreover, winners tend to

an individual's...

have a more proactive behavioural profile than losers [15-17], which points to agonistic experience, in adults at least, as a prime determiner of consistent inter-individual behavioural differences (“personality”) in crickets [16,18].

The main aim of the current investigation is, therefore, to determine if pre-adult agonistic experience during nymphal life has any lasting behavioural effects, which could account for different “personality” profiles in adults. Our results show that aggressive behaviour is far less fierce in nymphs, and that their interactions do not result in winner or losers effects typical for adults. However, adult males aggressively intimidate male nymphs, and we provide evidence that this influences their future adult behavioural profile.

this expression may be much better than referring to the *personality profile*. At least it needs to be made more clear how *personality* is defined.

Methods

Experimental animals and ethical note

Experimental animals, Mediterranean field crickets, *Gryllus bimaculatus* (de Geer), were taken from a breeding colony maintained at the animal housing facility of the University of Leipzig since 25 years. Under our standard conditions, they were kept from hatching to the first nymphal stage until adult in groups of 20-30 in transparent plastic boxes (35 x 19 cm and 30 cm high), with sand covered floor and egg cartons for shelter at 22–24°C, relative humidity 40–60%, 12 h: 12 h light: dark regime, with daily feeding on bran and fresh carrots, with water *ad libitum*. All experiments were performed in the months of March to June and October to December, at room temperature (20-24°C) during daylight hours, excluding midday and generally rainy, overcast days when aggression tends to be depressed [19,20]. All treatments conform to Principles of Laboratory Animal Care and German Law on Protection of Animals (*Deutsches Tierschutzgesetz*). A specific

license for experiments on invertebrates is not required in Germany. All animals were returned to the breeding stock after the experiment. Our analysis is based on observations of 1032 crickets, of which none were used more than once for an experiment.

For most experiments (Figs 1 - 5, and unless otherwise stated) we used male, last stage nymphs, and sexually mature adult males (>10 days after the final moult), that were taken from the standard breeding colony and kept socially isolated in individual glass jars for 48 h prior to experimentation, under the same ambient conditions with ample food and water ("short term isolates", STI).

Behavioural responses to conspecific contact and antennal stimulation

For these experiments (Figs 1 and 2), the behaviour of the test-crickets was observed in a clear Perspex-glass rectangular observation arena (l. w. h.: 16 × 9 × 7 cm) with a sand-covered floor. We recorded the responses to either contact with another individual of same or different sex and developmental stage (Fig 1), or to manually performed stroking of the antennae for ca. 20 s with the freshly severed antenna of a donor cricket, that was anaesthetised by cooling, and was either of the same or different sex and developmental stage (Fig 2). In one experimental series, the donor antenna of a mature adult male was washed twice for 10 min with n-hexane (Sigma Aldrich, Deisenhofen, Germany) to remove cuticular pheromones [21,22]. The following responses were recorded: *antennal fencing*, in which two individuals lash each other's antennae (also shown at the beginning of courtship between adults); *mandible threat display*, when an individual opens its mandibles; *attack*, when one individual lunges and physically engages an opponent; mandible spread angle: the angular extent of mandible

...observed and...

spreading. The latter was measured post event by analysing filmed responses (4K Video, Sony Alpha 6300 with Zeiss macro-zoom objective) on a computer (Dell Precision 3620, Round Rock, Texas, USA) with the software MB-Ruler (version 5.3, MB Softwaresolutions, Iffezheim, Germany).

Evaluation of aggression

Aggressive behaviour was evaluated in dyadic contests between two equally sized males (< 5% weight difference) in the observation arena (Figs 3, 4, 5 and 6D). The opponents were placed at opposite ends of the arena and separated by a sliding door in the middle. The animals' interactions were then noted when the door was removed. A scale of 0-6 was used to denote the level of aggressive escalation [20]: *Level 0*: mutual avoidance (no aggression). *Level 1*: one cricket attacks, the other retreats (pre-established dominance). *Level 2*: antennal fencing. *Level 3*: mandible threat display by one cricket. *Level 4*: mandible threat displays by both crickets. *Level 5*: mandible engagement. *Level 6*: grappling (an all-out fight). Contest duration was measured to the nearest second as the time from initial contact up until establishment of a loser and winner with a stopwatch, deducting pauses that occasionally occurred when the animals lost contact. Since nymphs appeared to be far less aggressive than adults, we tested the influence of various treatments and experiences known to enhance aggression in adults in order to reveal their aggressive potential:

colon or full stop?

Influence of winning and losing

In one set of experiments (Fig 3), pairs of adult males were first matched against each other to yield losers that retreat first and become submissive, and winners that generate

the rival song and become hyper-aggressive [11,12]. Maximally 10 mins later, winners were re-matched against winners, and losers against losers. We attempted the same procedure for male nymphs. However, their interactions did not always generate clear winners and losers, so we only evaluated those interactions where this seemed apparent (see results).

Results

Influence of prior antennal stimulation (*priming effect*)

Stimulation of an individual's antenna by manually stroking it with the freshly severed antenna of another adult donor male is known to increase aggression in adult males [23]. Here we compared the effect of prior antennal stimulation (20 s) on the aggression of adults and nymphs 5 min after losing a dyadic contest in a second contest against the previous winner 10 min later, using antennae donated from either male adults or nymphs (Fig 4).

make sentence more clear

Influence of the octopamine agonist chlordimeform coupled with priming

Since the priming effect is increased dramatically in adults treated with the tissue permeable, irreversible octopamine agonist chlordimeform hydrochloride (CDM; [23]), we tested this procedure on male nymphs (Fig 5A). Individual nymphs were first anaesthetised by cooling and then injected with 20 µl/ 1 mM CDM (Sigma Aldrich, Deisenhofen) dissolved in saline solution containing 1% DMSO (dimethylsulfoxide; saline components in mMol L^{-1}: NaCl 140, KCl 10, $CaCl_2$ 7, $NaHCO_3$ 8, $MgCl_2$ 1, N-trismethyl-2-aminoethanesulfonic acid 5, D-trehalose dehydrate 4, pH 7.4) using a micro-syringe inserted through the membranous cuticle underlying the pronotal shield. Control received vehicle only (DMSO in saline). After waiting for 50-60 min, test crickets

were subjected to antennal stimulation, as above, using an antenna from an adult male. Immediately afterwards, dyadic contests were staged between pairs of weight-matched treated nymphs.

Influence of food as a resource

Since resources such as food [24] promotes aggression in adult crickets, we tested whether its presence also influences aggression in nymphs. For this, male nymphs were deprived of food for 48 hours and their aggressiveness subsequently evaluated in dyadic contests staged in the immediate presence of freshly cut carrot (Fig 5B). Control contests were staged in the absence of food.

Influence of social experiences in nymphs on their behaviour when adult

To test for possible long-term behavioural effects of aggressive subjugation of nymphs by adults, we generated 3 cohorts of adult males housed under different social conditions (Fig 6A). One cohort was raised in the complete absence of adults as a group of 30-40 male nymphs from the moment of hatching ~~to the first nymphal stage~~ until adult and sexually mature. A second cohort was raised in the same way, except that 10 sexually mature adult males were introduced into the nymphal colony as soon as the majority had moulted to the 4th nymphal stage. The third cohort differed only in that 10 sexually mature adult females were introduced into the nymphal colony.

Five to six days after introducing the adults, when the nymphs were between the 5th and 6th nymphal stage, we visually recorded the number of either nymph-nymph or adult-nymph attacks (lunges with physical engagement involving pushing and/or biting) in the 3 colonies for ten 1 h observation periods, spaced 2 per day (09:00-10:00 and

adult

14:00-15:00), over 5 consecutive days. Other aspects of aggression could not be reliably evaluated under grouped conditions. Before each observation period, we first adjusted colony sizes to 30 nymphs (plus 10 males or females) by randomly removing nymphs if necessary. From the data gathered we calculated the mean number of attacks (and 95% CI) experienced per nymph per hour (Fig 6B).

Finally, individual nymphs were taken from each of the 3 colonies after moulting to the last nymphal stage, and kept socially isolated for at least 14 days until adult and fully sexually mature (long-term isolates, LTI). We then evaluated their general exploratory behaviour, as described below, and shortly after this their aggressiveness, as described above.

Evaluation of exploratory behaviour

To compare the exploratory behaviour of LTI adult crickets raised either in the absence or presence of adult males, individuals were placed in the observation arena and their activity was recorded for 5 min with a digital video camera (Basler acA1920-155uc, Ahrensburg, Germany, 60 frames/s) and stored on a computer (Dell Precision 3620, Round Rock, Texas, USA) for subsequent analysis with video-tracking software (EthoVision XT14, Noldus, Wageningen, Netherlands) as given in detail elsewhere [16]. In brief, the centre point of each cricket was detected by the grey scaling method (minimum grey value 0, maximal 140) and its movement tracked on an X-Y coordinate system. To reduce noise from the animal’s breathing movements, we set a “minimum distance moved” filter of 0.2 cm. For each animal we recorded the total distance moved as the length of the animals´ path covered in 5 min, and the mean walking velocity of bouts of actual walking, excluding times when the animal was stationary (Fig 6C).

Statistical analysis

All statistical tests were performed using commercial software (Prism 7, GraphPad Software Inc., La Jolla, CA, USA) running on a Macintosh computer (Apple Computers, Cupertino, CA, USA). Fisher´s exact test was performed to compare relative frequencies of behavioural elements between groups. The Shapiro-Wilk test was used to test for data normality. For normally distributed data we give the means and 95% confidence interval (CI) of the mean. Student´s unpaired t-test was used to test for significant differences between the means of two independent groups. For nominal data (level of aggression) and fight duration, which was not normally distributed even after log transformation, we calculated the median and interquartile range (IQR) and applied non parametric procedures to test for significant differences in data distributions: The Mann-Whitney U test for unpaired data sets and the Wilcoxon signed-rank test for paired data sets. The numbers of crickets for each experiment and test group are indicated in the figures and/or legends. The significance level alpha was set to $p < 0.05$ but in some cases (noted in text), a data set was compared to two other sets, so we applied the Bonferroni correction to alpha (= 0,025) to compensate for errors due to multiple comparisons.

decimal point

Results

Behavioural responses to conspecific contact

On contact, male adults typically behave aggressively towards conspecific male adults, but not towards females which they court and try to copulate. Accordingly, as a first step towards evaluating the aggressiveness of male nymphs, we evaluated their behavioural responses on initial contact to other individual nymphs and adults of both sexes, and

compared this with adult responses (Fig 1). As shown in Fig 1A, 50% of a total of 20 tested male nymphs showed antennal fencing behaviour towards conspecifics, regardless of their age group or sex. Male adults, in comparison, showed antennal fencing behaviour towards conspecifics, significantly more often and again indiscriminately of age-group or sex (90-95% Fisher's exact tests: $p = 0.0033$ for all comparisons, excepting responses towards female nymphs: $p = 0.0138$).

Male nymphs also showed the mandible threat display almost equally often towards both male nymphs (25%) and female nymphs (30%), but never towards male or female adults (0%; Fisher's exact tests for response towards adult females and males: $p = 0.0202, 0.0471$ respectively; latter insignificant after Bonferroni correction to $alpha = 0.025$). In comparison, male adults exhibited the mandible threat display significantly more frequently towards male nymphs (90%), male adults (90%) and female nymphs (75%; Fisher's exact tests: $p < 0.0005, < 0.0005, 0.0104$ respectively), but never towards adult females (0%, significantly different to response to female nymphs, Fisher's exact test: $p < 0.0005$). Similarly, male nymphs occasionally attacked both male (20%) and female nymphs (20%), but never adult males and females (0%; differences not, however, significant: Fisher's exact tests: both $p = 0.0305$, with alpha set to 0.025). Adults, in comparison, attacked other adult males (70%), male nymphs (90%) and female nymphs (80%) significantly more often than male nymphs (Fisher's exact tests: $p < 0.0005$ for each case with alpha set to 0.025). Male adults, however, never attacked adult females (0%, significantly different to response to female nymphs, Fisher's exact test: $p < 0.0005$ with alpha set to 0.025).

Fig 1. Agonistic actions of male nymphs and adults towards other nymphs and adults of both sexes. Bars giving the frequency of **(A)** antennal fencing, **(B)** mandible threat display and **(C)** attack (lunging with physical engagement) of STI male nymphs and adults towards different opponents (see key in C): male nymphs (light blue bars), male adults (blue bars), female nymphs (light red bars) and female adults (red bars; N = 20 for each group, always comprising different animals). Significant differences between groups are indicated (Fisher's exact test in A: * $p < 0.05$, ** $p < 0.01$; in B and C: * $p < 0.025$, *** $p < 0.0005$ after Bonferroni correction due to two comparisons).

Behavioural responses to antennal stimulation

Our data so far indicate that cricket nymphs do not discriminate between male and female nymphs and that they also exhibit mandible threat displays less often than adult crickets on confronting a conspecific, particularly an adult male (Fig 1). To analyse this further, we evaluated the response of male nymphs and adults to manual stimulation of their antenna using the excised antennae taken from male and female adults and male nymphs as donors (Fig 2A). Confirming an earlier study on adults [23], adult male crickets frequently exhibited mandible threats in response to stroking their antenna with an adult male antenna (80%). Compared to this, male nymphs exhibited mandible

hexane-washed

threats significantly less frequently (45%, Fisher's exact test: $p = 0.0024$ with alpha set to 0.025). In response to stimulation with a washed male antenna, adult males showed the mandible threat response less often (40%, Fisher's exact test: $p = 0.0034$), but now equally ~~as~~ often as male nymphs (40%). Similarly, male nymphs and adults showed an equal low incidence of mandible threats when stimulated with a male nymph's antenna (both 35%). Finally, in response to stimulation with an adult female antenna, male

nymphs showed essentially the same frequency of mandible threat displays (40%), whereas adult males responded significantly less often (5%, Fisher's exact test: $p = 0.0197$).

As shown in Fig 2B, male nymphs also spread their mandibles significantly less wide in response to stimulation with an adult male antenna than adults (nymphs: mean 40°, 95% CI: 36, 44, N = 18; adults: mean 71°, 95% CI: 69, 74, N = 32; Student's unpaired t-test: $p < 0.0005$, alpha set to 0.025). To test if this reflects some physical inability, we also measured the mandible spread angle during feeding (Fig 2B, right). This revealed that nymphs open their mandibles significantly wider when feeding (mean 69°, 95% CI: 66, 72, N = 18, Student's unpaired t-test: $p < 0,0005$, alpha set to 0.025), and equally wide as adults during both feeding (mean 72°, 95% CI: 68, 75, N = 18) and in response to antennal stimulation.

Fig 2. Mandible threat display in response to antennal stimulation. **(A)** Frequency of mandible threat for STI male adults and male nymphs (light and dark blue bars respectively) in response to mechanical stimulation with a freshly excised antenna from different donors (left to right): male adult, male adult and washed twice in n-hexane, male nymph and female adult. **(B)** Maximum angle of mandible spreading (mean and 95% CI) observed for male adults and nymphs in response to antennal stimulation (Ant. stim.) with a freshly excised male antenna and during feeding. Significant differences between data sets are indicated (A: Fisher's exact test, B: Student's unpaired t-test: * $p < 0.025$, ** $p < 0.005$, *** $p < 0.0005$; after Bonferroni correction due to two comparisons, N for each group is given in the bars).

Influences of winning and losing

Since the experience of winning and losing an aggressive interaction has a strong effect on the expression of aggression in adult crickets [3], we next tested whether ~~prior~~ agonistic experiences influence subsequent aggressive behaviour in STI ~~nymphs~~. As control (Fig 3A), and confirming earlier studies [11,12], the first fights between STI adult male crickets typically escalated to level 4 (Median, IQR = 4, 4-5, N = 20) and last 6 s (Median, IQR = 6 s, 4-10, N = 20). Compared to this, contests between pairs of adult crickets that experienced two successive wins at 5 min intervals escalated to a significantly higher level of aggression and the fights lasted longer (level: Median, IQR = 5, 5-6, duration: Median, IQR = 15 s, 10-19, N = 20, Wilcoxon signed-rank tests compared to 1st fight: $p_{level} = 0.0020$, $p_{duration} < 0.001$). Conversely, contests between losers of two consecutive fights were significantly less fierce than at the first fight (level: Median, IQR = 0, 0-1, duration: Median, IQR = 0 s, 0-1, N = 20, Wilcoxon signed-rank tests compared to 1st fight: $p_{level} < 0.001$, $p_{duration} < 0.001$).

These winner and loser effects were not evident for dyadic contests between weight-matched STI male nymphs (Fig 3B). Because the winners and losers of a match between nymphs are not always immediately apparent, we only evaluated interactions in which the establishment of dominance seemed clear, in that a “loser” retreated from a “winner” that in turn continued to lunge at the opponent. Compared to adults, contests between male nymphs were less fierce in that they never escalated above level 3 (Median, IQR = 2, 1-2, *N* = 20) or lasted longer than 4 s (Median, IQR = 2 s, 1-2, *N* = 20). Furthermore, winners of two previous interactions at 5 min intervals showed no significant change in their aggressiveness compared to the initial interaction (Wilcoxon signed-rank tests: $p_{level} > 0.9999$, $p_{duration} = 0.0625$). Similarly, interactions

between losers of two preceding contests were also not significantly different to those of the same animals at their first contest (Wilcoxon signed-rank tests: p_{level} = 0.0938, $p_{duration}$ = 0.2407).

Fig 3. Effects of aggressive experience on subsequent aggression. (A) Male adults. **(B)** Male nymphs (both STI)**.** Bars give the level (top) and duration (bottom) of agonistic interactions between pairs of STI winners (red bars) and pairs of losers (blue bars) from two previous contests, compared to their first interaction (1st fight, grey bars). Circles: median, boxes: interquartile range, whiskers: 10th and 90th percentiles, N = 20 for each bar. Significant differences between paired date sets are indicated (Wilcoxon signed-rank test: ** p < 0.01, *** p < 0.001). Note the absence of winner and loser effect in nymphs.

data

reword, mention and explain grey bars first, as in the legend!

Effect of prior antennal stimulation on fighting behaviour (*priming*)

We next tested whether nymphs show increased aggression after prior antennal stimulation as found previously for adults [23] and confirmed here. As shown in Fig 4A, STI adult losers of a first fight exhibit significantly reduced aggression when re-matched against their previous opponents at a second fight 10 mins later (Wilcoxon signed-rank tests: N = 20, p_{level} < 0.001, $p_{duration}$ < 0.001). This loser effect is, however, no longer evident in losers after stimulating their antennae with the freshly excised antenna of another adult male (Wilcoxon signed-rank tests: N = 20, p_{level} = 0.7327, $p_{duration}$ = 0.4359). Interestingly, we now found that this priming effect on adult loser aggression was not evident following mechanical stimulation with an antenna from a male nymph (Wilcoxon signed-rank tests: N = 20, p_{level} < 0.001, $p_{duration}$ < 0.001).

Contrary to adults, mechanical antennal stimulation of male nymphs (Fig 4B) after losing an initial contest failed to have any significant effect on their aggression expressed at a subsequent, second contest, regardless of whether an adult male antenna (Wilcoxon signed-rank tests: N = 20, p_{level} = 0.3398, $p_{duration}$ = 0.3029), or male nymph´s antenna (Wilcoxon signed-rank tests: N = 20, p_{level} = 0.1289, $p_{duration}$ = 0.4469) was used as a stimulus.

correct sequence? please check

Fig 4. Influence of prior antennal stimulation on subsequent aggression. Bars giving the level (top) and duration (bottom) of agonistic interactions between weight matched pairs of STI male nymphs (**A**) and adults (**B**) at their 1st interaction (light grey bars) and for the same animals 10 min later, before which the losers were either untreated (grey), or stimulated with an antenna from a male adult (red) or male nymph (light red). Circles: median, boxes: interquartile range, whiskers: 10th and 90th percentiles, N = 20 per group. Significant differences between paired data sets are indicated (Wilcoxon signed-rank test: *** p < 0.001).

rephrase, make more simple

Priming and the octopamine receptor agonist chlordimeform (CDM)

In a further attempt to promote aggression in STI nymphs, we next evaluated their interactions after treatment with the octopamine receptor agonist chlordimeform (CDM) coupled with the aggression priming stimulus (antennal stimulation with an antenna from an adult male, Fig 5A). In STI adults, this leads to a dramatic increase in the escalation level and duration of subsequent aggressive encounters [23]. In nymphs, however, we observed no significant change in escalation, but a just significant increase in contest duration (Mann-Whitney *U* tests: N = 13, p_{level} = 0.6267, $p_{duration}$ = 0.0425). Notably,

could this be due to the relative age of these nymphs?

however, one CDM-treated pair of nymphs showed actual physical fighting after prior antennal stimulation, during which the individuals interlocked their mandibles to push each other (level 5) in a contest that lasted 7 s, which is within the range typically exhibited by adults.

Fig 5. Effects of aggression modulators on aggression in male nymphs. Bars giving the level and duration of agonistic actions between STI male nymphs. **(A)** After treatment with the octopamine agonist chlordimeform (CDM in 1% DMSO, N = 13) combined with antennal stimulation (red hatched bars). **(B)** In the presence of food (orange hatched bars, N = 20). Controls (non-hatched bars) received DMSO in saline (A, N = 13) or no food (B, N = 20). Significant differences between unpaired data sets are indicated (Mann-Whitney *U* test: * $p < 0.05$).

Food fights

Earlier descriptions of competition in cricket nymphs were based in part on observing their interactions in the presence of food [7]. Since such resources heighten aggression in adult crickets [24,25], we tested whether the presence of food increased aspects of aggression in nymphs as measured by us (Fig 5B). This revealed no significant differences in the level of escalation or contest duration between pairs of STI nymphs that interacted in the absence and presence of food (Mann-Whitney *U* tests: N = 20, $p_{level} = 0.9081$, $p_{duration} = 0.5490$).

what do you mean: groups of thirty or singe nymphs?

Influence of social experiences in nymphs on their behaviour when adult

Since our data reveal that an adult male will attack a nymph on contact in the small fighting arena (Fig 1C), we next compared how often this occurs in colonies of 30 male nymphs raised either in the absence of adults, or together with 10 sexually mature adult males or females (Fig 6A). As shown in Fig 6B, nymphs raised by their own ~~without adults~~ were rarely attacked by other nymphs under the grouped culture conditions (mean attacks per nymph per hour for n = 10 x 1 h sessions: 0.07, 95% CI: 0.06, 0.08). Similarly, male nymphs housed with adult females were also hardly ever attacked by them (mean attacks per nymph per hour: 0.07, 95% CI: 0.04, 0.09). Contrasting this, individual nymphs housed with adult males were attacked by an adult almost once an hour, and significantly more often than by other nymphs or adult females (mean 0.93, 95% CI: 0.84, 1.02; Student's unpaired t-test: $p < 0.0005$ for both, with alpha set to 0.025) full stop

With respect to general motility, video-tracking analysis revealed that adult males raised without any contact to adult males ~~during development~~ were more proactive than those housed as nymphs together with adult males (Fig 6C). LTI adult male crickets that never had any social contact to adult males, walked a significantly greater total distance during a 5 min observation period (mean 189 cm, 95% CI: 174, 204, N = 20), compared to those subjected to potential subjugation by adult males as nymphs (mean 150 cm, 95% CI: 138, 162, N = 20; Student's unpaired t-test: $p < 0.001$). Furthermore, the mean velocity of the walking episodes was significantly faster for crickets from the pure nymph colony (mean 0.74 cm/s, 95% CI: 0.67, 0.81, N = 20), compared to ~~nymphs from~~ colonies with adult males (mean 0.54 cm/s 95% CI: 0.49, 0.59, N = 20; Student's unpaired t-test: $p < 0.001$)

Finally, an evaluation of aggression revealed that LTI adults from the pure nymph

colony, ~~that had never experienced contact to adult males~~, were significantly more aggressive than those from the mixed nymph-adult colony (Fig 6D, Mann-Whitney *U* tests: N = 20, p_{level} = 0.0337, $p_{duration}$ = 0.0223).

Fig 6. Influence of social experiences in nymphs on their behaviour when adult. **(A)** Schema of culture conditions: 30 male nymphs were housed either without adults (light blue) from hatching until the last nymphal stage, or with 10 adult males (blue) or 10 adult females (red) from the 4th to the last nymphal stage. **(B)** Bars giving the mean (± 95% CI) number of attacks experienced per nymph per hour for 10 x 1 h sessions, from either other nymphs in the pure nymph culture (light blue, number divided by 3 to adjust for the larger number of nymphs in the cage), or adult males (dark blue) or adult females (red) in the mixed cultures. **(C)** Bars giving the distance moved (left) and mean velocity (right) of walking bouts during 5 min (means ± 95% CI, N = 20 per group) for LTI mature adult males taken >14 days previous as last stage nymphs from the pure nymph colony (light blue) or the mixed nymph-adult male colony (blue). **(D)** As for C, but giving the level (left) and duration (right) of fights between pairs of crickets from the two groups (circles: median, boxes: interquartile range, whiskers: 10th and 90th percentiles). Significant differences are indicated (B and C: Student's unpaired t-test: *** $p < 0.0005$ for B after Bonferroni correction due to two comparisons; $p < 0.001$ for C. D: Mann-Whitney U test: * $p < 0.05$).

Discussion

may be explain a bit more

Agonistic experience, and in particular social defeat, is becoming recognised as a major factor that induces symptoms of severe depression and shapes consistent inter-individual differences in behaviour ("personality") in numerous animal groups [1,2]. In this paper we analysed the consequences of pre-adult (nymphal) agonistic experiences in crickets, *Gryllus bimaculatus*, which have become a model system for investigating the neurochemical control of aggression [3]. Earlier reports imply that aggression exhibited between male, last instar cricket nymphs is essentially the same as that between adult males, and has consequences for adult fitness, at least in terms of body size and survival rate [7]. Our study, however, which compares aggression in nymphs and adults, with a focus on how it influences future behaviour, calls for a different interpretation.

We show that agonistic interactions between weight-matched male nymphs are far less fierce than those between mature adult males. comma, ~~Nymph-nymph interactions are~~ typified by a significantly lower level of aggressive escalation and duration (Fig 3), ~~such that~~ Actual... actual physical fights were, ~~under normal circumstances,~~ never ~~ever~~ observed (see however below). This observation is ~~basically~~ not so different to that of Simmons ([26]), who noted that nymphs do not show grappling and flipping behaviour typical for adults [26], which would correspond to our level 5 and 6 fights. Of more relevance to the present study, ~~and as also discussed below~~, competition between nymphs does not result in winner and loser effects (Fig 3B), which in adults influence~~s~~ the intensity of subsequent aggressive interactions and the establishment of dominant-subordinate relationships with additional consequences for the expression of other behaviours [16].

Our findings raise the question why nymphs do not show the all-out fighting

characteristic for mature adult males? Most studies of hemimetabolous insects, including locusts and crickets, indicate that the central nervous apparatus underlying adult-specific behaviours such as flight, copulation and phonotaxis are already functional in nymphs [27-30], but cannot be normally recruited by the natural releasing stimulus [27]. We propose that the same applies to aggression. Firstly, and as also noted by Simmons [7], male nymphs that contact each other clearly exhibit some aspects of adult aggressive behaviour, including antennal fencing, mandible threat displays and lunging, but far less often (Fig 1). Furthermore, even though nymphs do not spread their mandibles as wide as adults on confronting another male, they are fully able to do so, at least during feeding (Fig 2B). Reduced mandible spreading in nymphs is also unlikely to be a cause of reduced aggressiveness, since neither mandible quality [31] nor the ability to use them [32], has any influence on contest intensity or duration. Hence, cricket nymphs appear to be ~~both motorically and physically as~~ capable as adults of exhibiting actual fighting behaviour, they only seem less inclined to do so ~~than adults~~.

they certainly will be weaker

The most parsimonious explanation for the limited aggressiveness of nymphs is that their ability to ~~generate and~~ respond to the natural aggression-releasing stimulus is not fully developed. In adult crickets, antennal fencing behaviour between males on first contact involves mechanical stimulation with transfer of male-specific semiochemicals that are essential for initiating normal aggression [21-23,33-36]. Notably, and contrasting the central nervous system [37], the sensory system of hemimetabolous insects undergoes dramatic changes during post-embryonic development, including the strengthening of synaptic contacts of existing afferents and the birth of numerous new ones with each moult [38]. Such changes in olfactory receptor neurones during development in locusts are known to result in increased chemo-afferent convergence on

the antennal lobe with maturation [39]. Our experiments suggest a corresponding scenario in crickets. In contrast to adults, nymphs do not discriminate between male and female nymphs since they show aggressive actions such as mandible threats and attacks almost equally towards both (Fig 1). Adult males also show these same elements of aggression on contacting both male and female nymphs, but practically never on contacting an adult female. Furthermore, about half of the tested male nymphs exhibited mandible threat displays in response to antennal stimulation with a severed antenna from a donor cricket, irrespective of whether the donor was a male nymph, male adult or female adult. Adult males, in contrast, show mandible threats significantly more often in response to stimulation with an adult male antenna (80%) and less often (5%) when the antenna was from a female, but showed similar response frequency as nymphs when a male nymph's antenna, or a washed adult male antenna was used (35-50%; Fig 2A). Additionally, adult losers become more aggressive after stimulation with an adult male antenna, but not in response to a male nymph's antenna (Fig 4).

kind of repetitive to results, please summarise

Together, these data suggest that cricket nymphs neither produce nor respond fully to adult contact pheromones. While differences in pheromonal signatures between the sexes are clearly documented in adult crickets (*Teleogryllus ocanicus*: [35]), studies of insect pheromones in nymphs are rare (e.g. [40]) and to our knowledge none focus on pheromones ~~those~~ controlling social behaviours, such as aggression. There is only evidence for developmental changes in the quality and quantity of cuticular hydrocarbons during adulthood [21].

In adults~~,~~ insects male-male contact leads to release of ~~the amine~~ octopamine in crickets [33] and to direct excitation of octopamine containing neurones by specific pheromone sensitive receptors in *Drosophila* [41]. While octopamine is not essential for the

expression [20] or initiation [23] of aggression, it increases the propensity to escalate and persist longer in fighting by raising an individual's threshold to flee [3]. Thus, a wide variety of experiences that promote aggression, including prior physical exertion (flying, fighting), antennal contact with another adult male's antenna, burrow residency, the presence of an adult female or food and winning a previous encounter, are ~~each~~ mediated by the neuromodulatory action of octopamine [42,43]. Contrary to adults, however, neither winning (Fig 3), stimulation with an adult male's antenna (Fig 4), nor the presence of food (Fig 5) led to any significant increase in aggression expressed by nymphs. This difference ~~to adults~~ could reflect developmental immaturity in both the pheromonal (see above) and octopamine systems. For example, adult crickets (female *Acheta domesticus*, males not tested) contain 50% more octopamine in their brains than last instar nymphs [44] and octopaminergic stimulation of the second messenger adenylate cyclase is more potent in the brain of adult locusts compared to nymphs [45]. Supporting this octopamine deficiency idea, experimentally increased octopamine signalling could compensate, at least partially, for immaturity in the olfactory, aggression-releasing pathway of nymphs. Thus, prior stimulation with an adult male's antenna coupled with application of the tissue permeable octopamine-receptor agonist chlordimeform (CDM), significantly increased the duration of contests between nymphs (Fig 5A). One treated pair even exhibited physical engagement with their mandibles (level 5) in a contest that lasted several seconds, shown otherwise only by adults. This observed exception supports our proposal that the central motor circuitry underlying male aggressive behaviour is in principle fully functional in the last stage cricket nymphs.

section again a bit repetitive of results

A main focus of this study was to determine the extent to which agonistic experience in nymphs influences their future behaviour. Winning and losing experiences

are known to change future aggression and generally proactive behaviours in numerous animal groups [2,10] including crickets [11,12,15,16]. Interactions between male nymphs, however, do not always generate clear winners and losers, and even in cases when this was reasonably clear, winners were not subsequently more aggressive, nor were the losers less so. Hence, nymph-nymph competition appears to have no influence on their future behaviour. Due to the dependence of the winner effect on octopamine in adults [11], we suspect that it's lack in nymphs may also reflect immaturity in the male-pheromone / octopamine signalling pathway. The lack of a loser effect is likely to have a similar, although somewhat different cause. In adults, retreat and resulting loser depression occurs in response to the aversive actions of the opponent during fighting [32], which causes activation of the nitric oxide signalling pathway [46].

Whereas nymph-nymph competition appears to have ~~little or~~ no influence on subsequent behaviour, our study revealed that adult males aggressively intimidate male nymphs with long lasting behavioural consequences. In a one to one situation, male adults attack nymphs as often as they attack adult males (Fig 1) and under grouped conditions with 30 male nymphs and 10 adult males, each nymph is attacked on average about once an hour (Fig 6B). In adults, multiple defeats (6) at one hour intervals induces a long-term depression of aggression which can last for days [13,14]. Here we now found that adults raised as nymphs with adult males are significantly less aggressive when tested around 2 weeks after their last social contact than adult males raised in the absence of adults (Fig 6D). Adults raised as nymphs with adult males are also less proactive in their behaviour than adults raised in their absence (Fig 6C). Hence, the experience of social subjugation by adult males during nymphal development can lead to life-long depression of both aggression and proactive

again quite repetitive of results, please shorten

comma,

, comma

Is this the same for male and females nymphs?

this sentence is correct, but complicated, can you rephrase?

behaviours. In adults, the depressing effect of chronic social defeat on adult behaviour is mediated by nitric oxide and serotonin [14], so that we are currently addressing the role of these neuromodulators in establishing individual behavioural profiles during development. We conclude that social contact to adult males during pre-adult life is a major factor forging consistent, life-long, inter-individual behavioural differences between adults, i.e. animal “personality”.

please explain this term a bit more, here it always appears as a key word thrown into the text, without a critical evaluation and referencing

Acknowledgements

This manuscript forms part of the doctoral thesis of JSB. We thank Jan Rillich for helpful comments.

REFERENCES

1. de Boer SF, Buwalda B, Koolhaas JM. Aggressive Behavior and Social Stress. In G. Fink (Ed.), Stress: Concepts, Cognition, Emotion, and Behavior. 2016; pp. 293-303. Burlington: Academic Press. doi: https://doi.org/10.1016/B978-0-12-800951-2.00036-4

2. Briffa M, Sneddon LU, Wilson AJ. Animal personality as a cause and consequence of contest behaviour. Biol. Lett. 2015;11(3). doi: https://doi.org/10.1098/rsbl.2014.1007

3. Stevenson PA, Rillich J. Fight or flee? Lessons from insects on aggression. Neuroforum. 2019*; 25*, 3-14. doi: https://doi.org/10.1515/nf-2017-0040

4. Wommack JC, Taravosh-Lahn K, David JT, Delville Y. Repeated exposure to social stress alters the development of agonistic behavior in male golden hamsters. Hormones and Behavior. 2003; 43(1), 229-236. doi: https://doi.org/10.1016/s0018-506x(02)00029

5. Caldeira V, Woodin EM. Childhood exposure to aggression and adult relationship functioning:Depression and antisocial behavior as mediators. Journal of Family Violence. 2012; 27(7), 687-696. doi: https://doi.org/10.1007/s10896-012-9453-1

6. Arseneault L. The long-term impact of bullying victimization on mental health. World Psychiatry. 2017; 16:27-8. doi: https://doi.org/10.1002/wps.20399

7. Simmons LW. Competition between nymphs of the field cricket, *Gryllus bimaculatus* (Orthoptera: Gryllidae) and its effects on some life-history components of fitness. Journal of Animal Ecology, 1987;*56*, 1015-1027

8. Niemela PT, Vainikka A, Hedrick AV, Kortet R. Integrating behaviour with life history: boldness of the field cricket, *Gryllus integer*, during ontogeny. Functional Ecology. 2012; 26(2), 450-456. doi: https://doi.org/10.1111/j.1365-2435.2011.01939.x

9. Han CS, Tuni C, Ulcik J, Dingemanse NJ. Increased developmental density decreases the magnitude of indirect genetic effects expressed during agonistic interactions in an insect. Evolution. 2018. doi: https://doi.org/10.1111/evo.13600

10. Hsu Y, Earley RL, Wolf LL. Modulation of aggressive behaviour by fighting experience: mechanisms and contest outcomes. Biol Rev Camb Philos Soc. 2006; *81*(1), 33-74. doi: https://doi.org/10.1017/S146479310500686X

11. Rillich J, Stevenson PA. Winning fights induces hyperaggression via the action of the biogenic amine octopamine in crickets. PLOS ONE*.* 2011; *6*, e28891. doi: https://doi.org/10.1371/journal.pone.0028891

12. Stevenson PA, Rillich J. Isolation associated aggression - a consequence of recovery from defeat in a territorial animal. PLoS One*.* 2013; *8*(9), e74965. doi: https://doi.org/10.1371/journal.pone.0074965 PONE-D-13-14351 [pii]

13. Rose J, Rillich J, Stevenson PA. Chronic social defeat induces long-term behavioral depression of aggressive motivation in an invertebrate model system. PloS ONE. 2017b; 12(9):e0184121, 1-16. doi: https://doi.org/10.1371/journal.pone.0184121

14. Rillich J, Stevenson PA. Serotonin mediates depression of aggression after acute and chronic social defeat stress in a model insect. Front Behav Neurosci. 2018; 12, 233. doi: https://doi.org/10.3389/fnbeh.2018.00233

15. Kortet R, Hedrick A. A behavioural syndrome in the field cricket *Gryllus integer*: intrasexual aggression is correlated with activity in a novel environment. Biological Journal of the Linnean Society. 2007; 91(3), 475-482. doi: https://doi.org/10.1111/j.1095-8312.2007.00812.x

16. Rose J, Cullen DA, Simpson S, Stevenson PA. Born to win or bred to lose: aggressive and submissive behavioural profiles in crickets. Animal Behaviour. 2017a; 123, 441-450. doi: https://doi.org/10.1016/j.anbehav.2016.11.021

17. Abe T, Fujiyama N, Tomimatsu H, Nagayama T. Age-dependent and social status-dependent behavioural plasticity of the cricket *Gryllus bimaculatus*. Animal Behaviour. 2018; 141, 1-7. doi: https://doi.org/10.1016/j.anbehav.2018.05.003

18. Jäger HY, Han CS, Dingemanse NJ. Social experiences shape behavioral individuality and within-individual stability. Behavioral Ecology. 2019. doi: https://doi.org/10.1093/beheco/arz042

19. Dixon KA, Cade WH. Some factors influencing male-male aggression in the field cricket *Gryllus integer:* time of day age weight and sexual maturity. Animal Behaviour. 1986; *34*, 340-346

20. Stevenson PA, Hofmann HA, Schoch K, Schildberger K. The fight and flight responses of crickets depleted of biogenic amines. Journal of Neurobiology. 2000; 43(2), 107-120

21. Nagamoto J, Aonuma H, Hisada M. Discrimination of conspecific individuals via cuticular Pheromones by males of the cricket *Gryllus bimaculatus*. Zoological Science. 2005; 22(10), 1079-1088.

22. Iwasaki M, Katagiri C. Cuticular lipids and odors induce sex-specific behaviors in the male cricket *Gryllus bimaculatus*. Comparative biochemistry and physiology. Part A, Molecular & integrative physiology. 2008; 149(3), 306-313. doi: https://doi.org/10.1016/j.cbpa.2008.01.008

23. Rillich J, Stevenson PA. Releasing stimuli and aggression in crickets: octopamine promotes escalation and maintenance but not initiation. Frontiers in Behavioral Neuroscience. 2015; 9. doi: https://doi.org/10.3389/fnbeh.2015.00095

24. Nosil P. Food fights in house crickets, *Acheta domesticus*, and the effects of body size and hunger level. Canadian Journal of Zoology Revue Canadienne de Zoologie. 2002; 80(3), 409-417. doi: https://doi.org/10.1139/z02-018

25. Rillich J, Schildberger K, Stevenson PA. Octopamine and occupancy - an aminergic mechanism for intruder-resident aggression in crickets. Proceedings of the Royal Society London B. 2011; 278, 1873-1880. doi: https://doi.org/10.1098/rspb.2010.2099

26. Simmons, LW. Inter-male competition and mating success in the field cricket, *Gryllus bimaculatus* (de Geer). Animal Behaviour. 1986*; 34*, 567-579.

27. Stevenson PA, Kutsch W. Demonstration of functional connectivity of the flight motor System in all stages of the locust. Journal of Comparative Physiology A. 1988; 162(2), 247-259

28. Sakai M, Katayama T, Taoda Y. Postembryonic development of mating behavior in the male cricket *Gryllus bimaculatus* DeGeer. J.Comp.Physiol.[A]. 1990; 166, 775-784.

29. Kutsch W, Pener MP. Muscle activity underlying sexual behaviour in male locusts (*Locusta migratoria*) after normal and hormonally disturbed development. Physiological Entomology 1997; *22*(1), 47-54

30. Staudacher EM. The auditory system of last instars in *Gryllus bimaculatus* DeGeer. Physiological Entomology. 2009; 34(1), 18-29. doi: https://doi.org/10.1111/j.1365-3032.2008.00647.x

31. Judge KA, Bonanno VL. Male weaponry in a fighting cricket. PloS one. 2008; 3(12), e3980. doi: https://doi.org/10.1371/journal.pone.0003980

32. Rillich J, Schildberger K, Stevenson PA. Assessment strategy of fighting crickets revealed by manipulating information exchange. Animal Behaviour. 2007; 74(4), 823-836. doi: https://doi.org/10.1016/j.anbehav.2006.11.022

33. Adamo SA, Hoy RR. Agonistic behavior in male and female field crickets, *Gryllus bimaculatus*, and how behavioural context influences its expression. Animal Behaviour. 1995; 49(6), 1491-1501

34. Hofmann HA, Schildberger K. Assessment of strength and willingness to fight during aggressive encounters in crickets. Animal Behaviour. 2001; 62 (Part 2), 337-348

35. Thomas ML, Simmons LW. Sexual dimorphism in cuticular hydrocarbons of the Australian field cricket *Teleogryllus oceanicus* (Orthoptera: Gryllidae). J Insect Physiol. 2008; 54(6), 1081-1089. doi: https://doi.org/10.1016/j.jinsphys.2008.04.012

36. Sakura M, Aonuma H. Aggressive behavior in the antennectomized male cricket *Gryllus bimaculatus*. J Exp Biol. 2013; 216(Pt 12), 2221-2228. doi: https://doi.org/jeb.079400 [pii]10.1242/jeb.079400

37. Raper JA, Bastiani M, Goodman CS. Pathfinding by neuronal growth cones in grasshopper embryos. I. Divergent choices made by the growth cones of sibling neurons. J Neurosci. 1983; 3(1), 20-30

38. Pflüger HJ, Hurdelbrink S, Czjze A, Burrows M. Activity-dependent structural dynamics of insect sensory fibers. J. Neurosci.1994; 14, 6946-6955

39. Anton S, Ignell R, Hansson BS. Developmental changes in the structure and function of the central olfactory system in gregarious and solitary desert locusts. Microsc Res Tech. 2002; 56(4), 281-291. doi: https://doi.org/10.1002/jemt.10032

40. Thibert J, Farine JP, Cortot J, Ferveur JF. *Drosophila* food-associated pheromones: Effect of experience, genotype and antibiotics on nymphal behavior. PLoS One. 2016; 11(3), e0151451. doi: https://doi.org/10.1371/journal.pone.0151451

41. Andrews JC, Fernandez MP, Yu Q, Leary GP, Leung AK, Kavanaugh MP, Certel SJ. Octopamine neuromodulation regulates Gr32a-linked aggression and courtship pathways in *Drosophila* males. PLoS Genet. 2014; 10(5), e1004356. doi: https://doi.org/10.1371/journal.pgen.1004356

42. Stevenson PA, Rillich J. Controlling the decision to fight or flee: the roles of biogenic amines and nitric oxide in the cricket. Current Zoology. 2016; 62(3), 265-275. doi: https://doi.org/ 10.1093/cz/zow028

43. Rillich J, Rillich B, Stevenson PA. Differential modulation of courtship behavior and subsequent aggression by octopamine, dopamine and serotonin in male crickets. Hormones & Behavior. 2019. doi.org/10.1016/j.yhbeh.2019.06.006

44. Woodring JP, Meier OW, Rose R. Effect of development, photoperiod and stress on octopamine levels on the house cricket, *Acheta domesticus*. Journal of Insect Physiology. 1988; 34, 759-765.

45. Hiripi LS, Rózsa K. Octopamine and dopamine sensitive adenylate cyclase in the brain of Locusta migratoria during its development. Cell.Mol.Neurobiol. 1984; 4, 199-206

46. Stevenson PA, Rillich J. Adding up the odds - Nitric oxide signaling underlies the decision to flee and post-conflict depression of aggression. Sci Adv. 2015; 1(2), e1500060. doi: https://doi.org/10.1126/sciadv.1500060

Fig. 1

Click here to download Figure Fig 1.tif

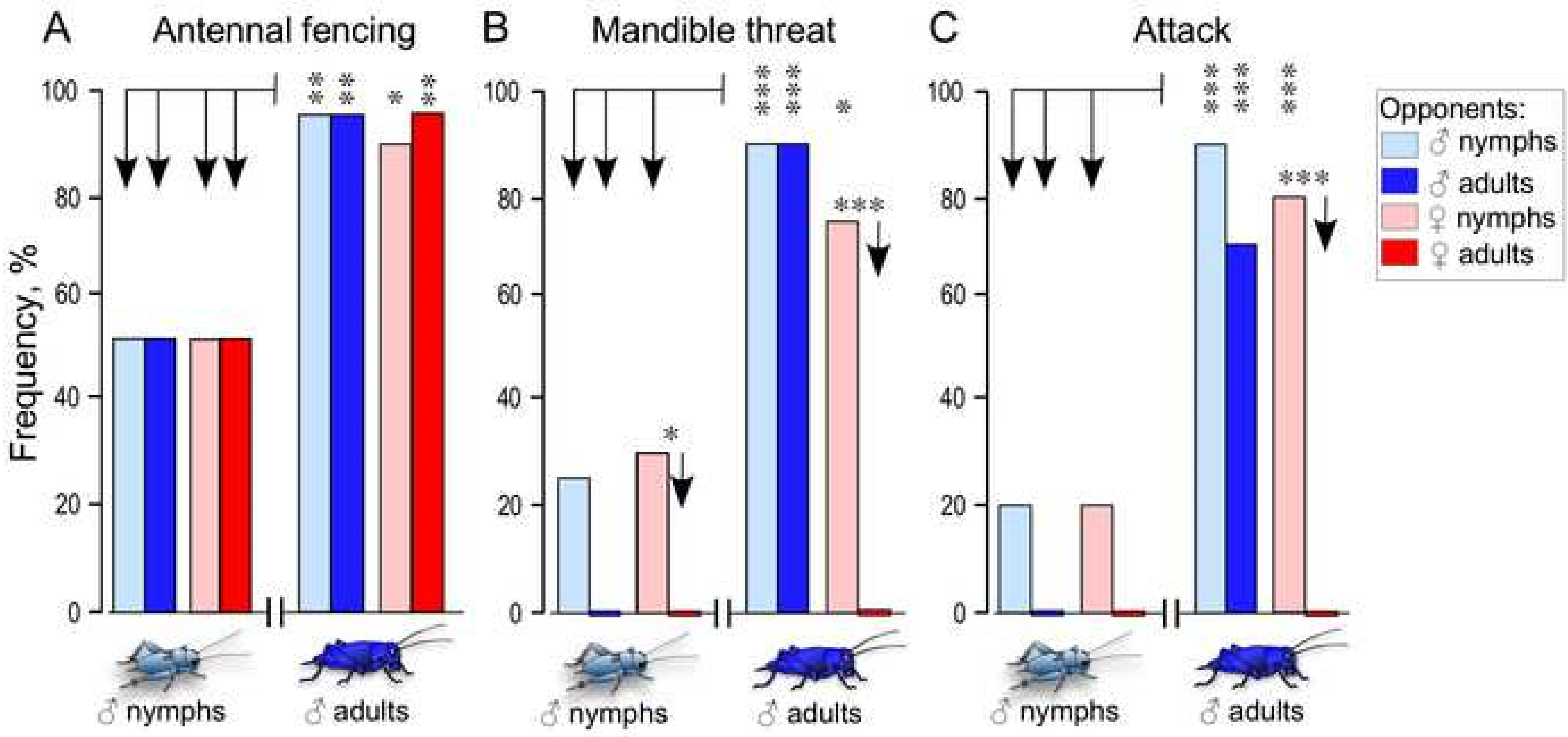

Fig. 2

Click here to download Figure Fig 2.tif

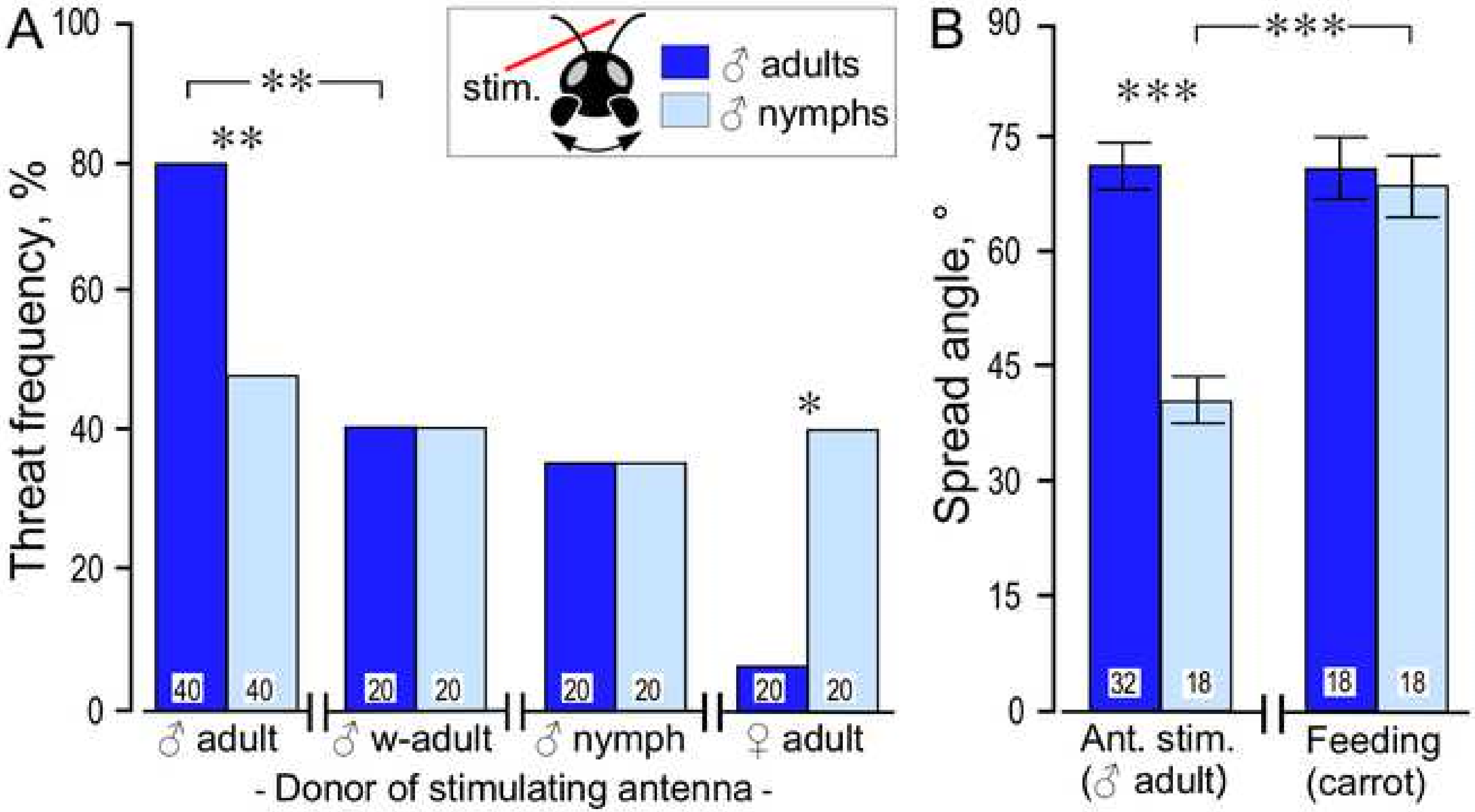

Click here to download Figure Fig 3.tif

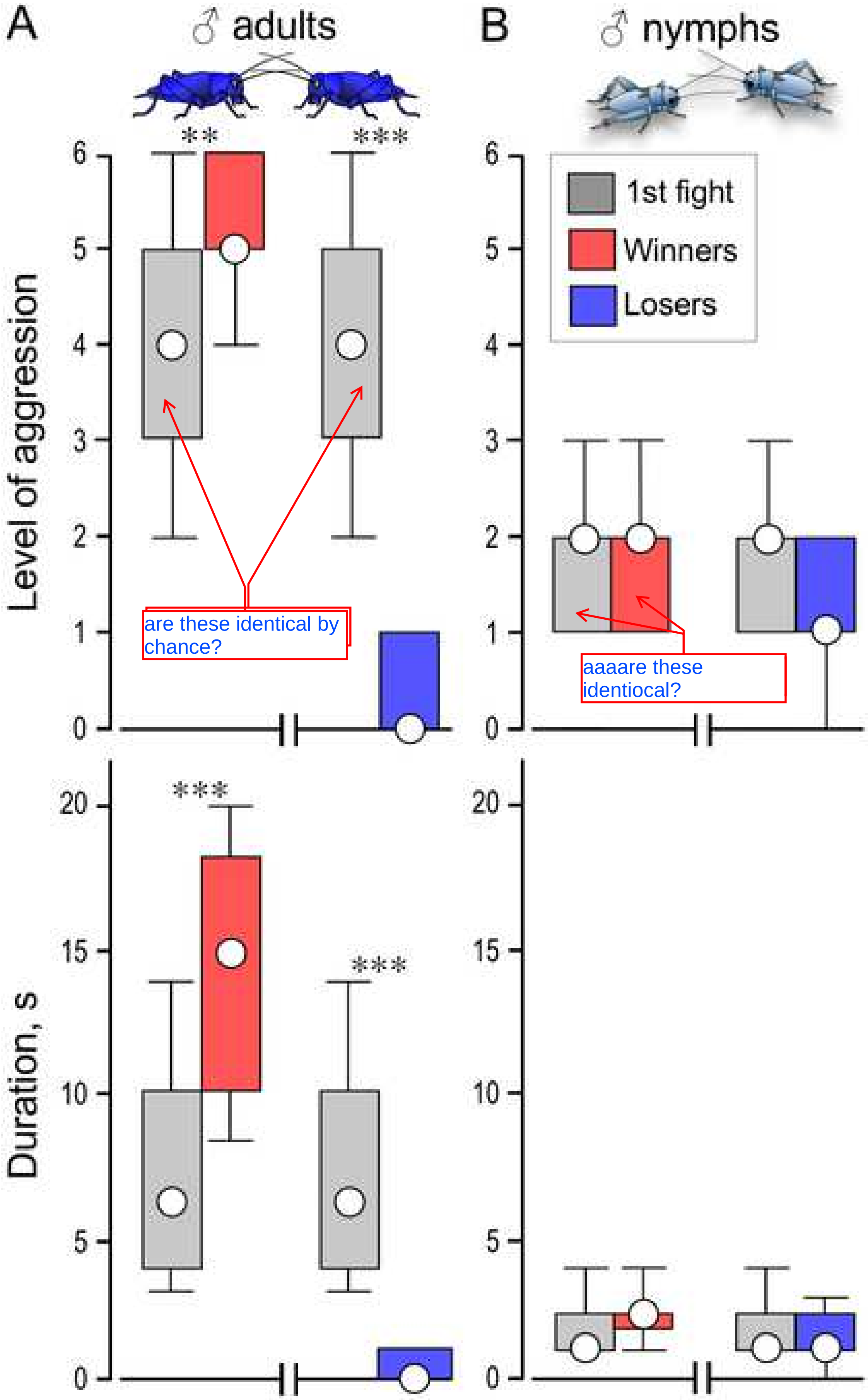

Fig. 4

Click here to download Figure Fig 4.tif

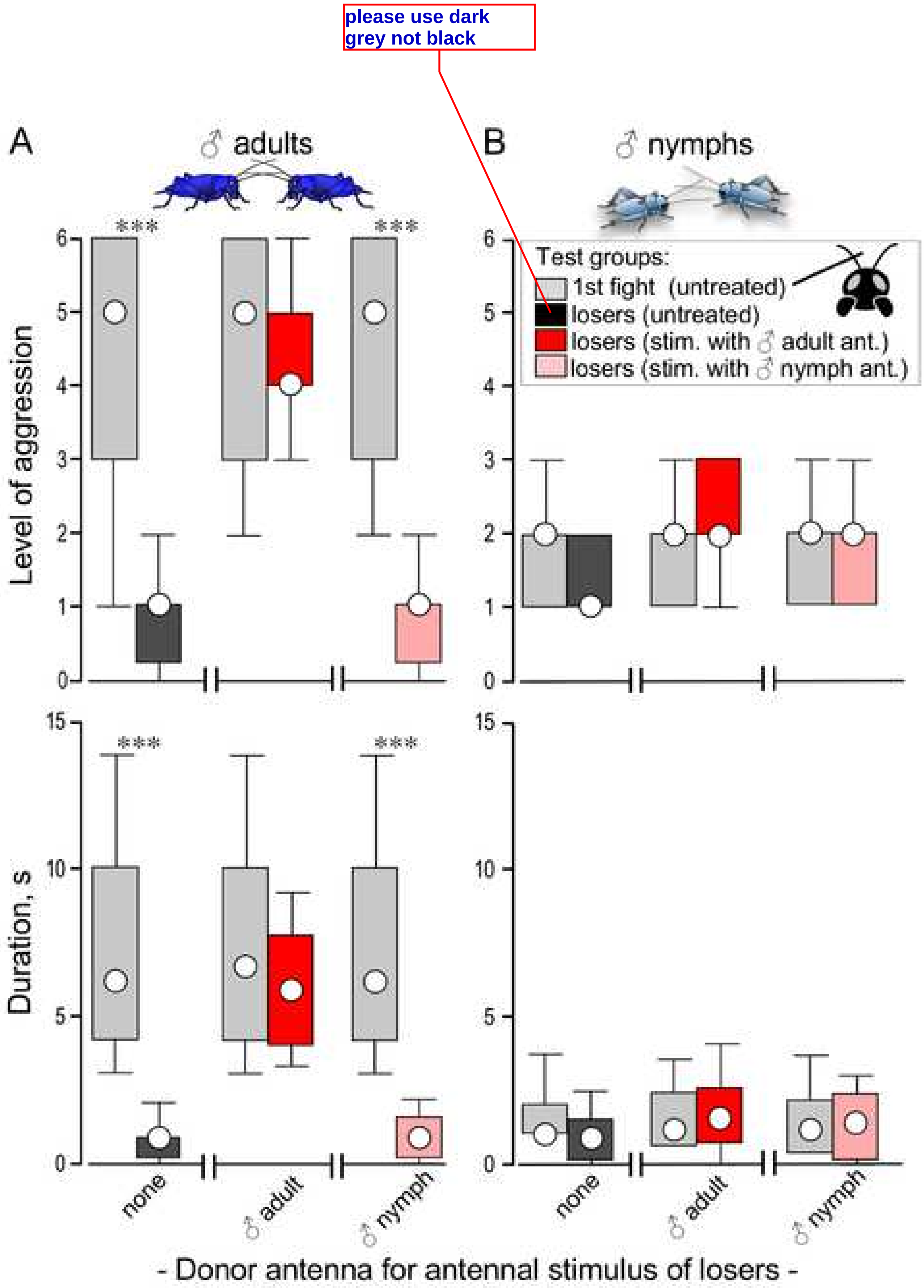

Fig. 5

Click here to download Figure Fig 5.tif

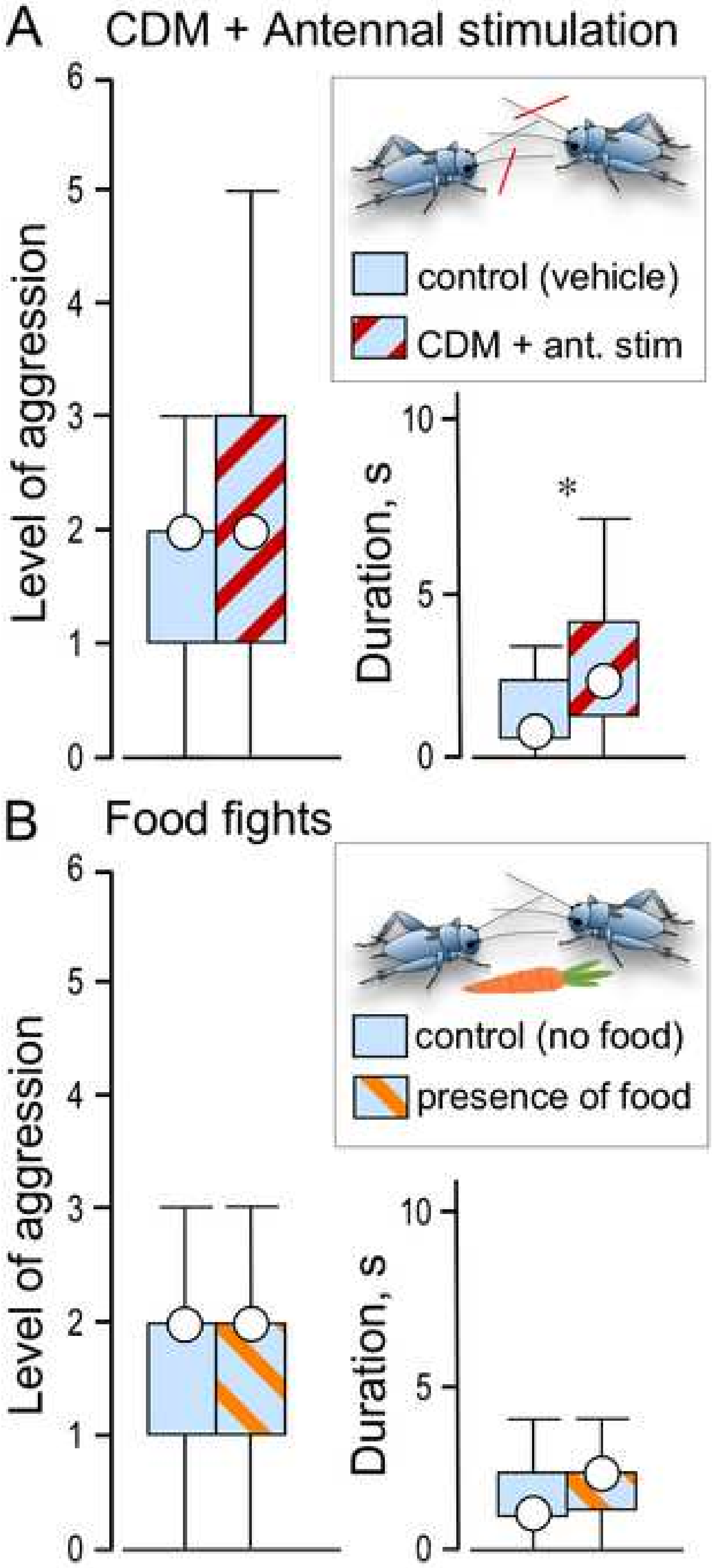

Fig. 6

Click here to download Figure Fig 6.tif

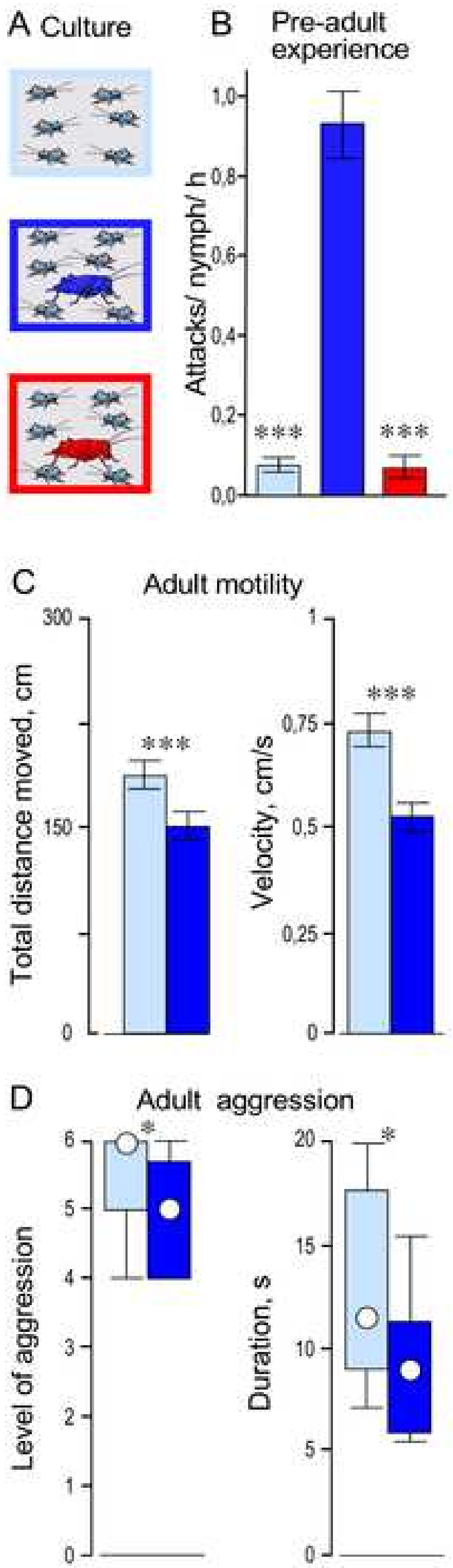

Supplement: Attachment — Submitted filename: ref04-PONE-D-19-35154_reviewer.pdf [file pone.0230743.s002.pdf]
