# Supplementary material for: Pre-adult aggression and its long-term behavioural consequences in crickets
Source: PLoS One. 2020 Mar 26;15(3):e0230743. doi: 10.1371/journal.pone.0230743 (PMC7098602; doi:10.1371/journal.pone.0230743)
Supplement: Attachment — Submitted filename: Response to Reviewers.docx [file pone.0230743.s003.docx]

**Response to Reviewers (PONE-D-19-35154)**

**Reviewer 1**

**1) Fig 1 and 2 examine behavioral responses to conspecific contact and antennal**

**stimulation. The results here are categorized in and recorded as: antennal fencing, in**

**which two individuals lash each other’s antennae; individual opens its mandibles;**

**attack, when one individual lunges and physically engages an opponent; mandible**

**spread angle: the angular extent of mandible spreading. It is not clear in the**

**presentation of the results as to why this battery of behaviors are the most adequate.**

We have changed the heading of the first section of Results to: “Agonistic responses of

nymphs and adults to each other’s presence” and added the following in results: “Since

antennal fencing is required to initiate fighting [26], and the mandible threat display

signals aggressive intent, while lunging with physical engagement (“attack”) is a clearly

aggressive action, we based this analysis on these three behaviours (Fig 1).

We have also added the following in Method (Evaluation of aggression): “As described

in detail elsewhere [4,20], aggressive contests in crickets are characterised by a highly

stereotyped series of increasingly aggressive actions, and can be scored on a simple

scale (0 – 6) denoting the “level of aggression” to which the animals escalate.”

**2) Mechanistically, it is unclear how the experiments on the influence of prior antennal**

**stimulation (priming effect) were done and how these results are necessary in addition to the priming of aggression (this sounds like a positive control)?**

As also suggested by another referee, we have amended the Methods (Influence of

prior antennal stimulation) to make our procedure more clear: "We first staged

contests between pairs of adults and between pairs of nymphs. The antennae of each

resultant loser was then stimulated (20 s, 5 min post defeat) with the antennae donated

from either a male adult or a male nymph. Each loser’s aggressiveness was then

evaluated 10 min later in a second contest against the previous winner (Fig 4).” Please

note, that the priming experiments were performed to see if prior antennal stimulation

increases aggression in nymphs, as shown for adults. To clarify this, we now state in

Methods: “Since nymphs appeared to be far less aggressive than adults, we tested the

influence of the following various treatments known to enhance aggression in adults in

order to gain an indication of the maximal aggressive capacity of nymphs, and possible

causes for it being less than in adults.

**3) Authors suggest that these decrease is explained by the possibility that nymph's**

**ability to generate and respond to the natural aggression-releasing stimulus is not fully developed. .…. This difference to adults could reflect developmental immaturity in both the pheromonal and octopamine systems.**

We thank the referee for his positive comments here. To make our arguments even

clearer we have rearranged the section in the discussion, and conclude as suggested

with: “In conclusion, we propose that the reduced aggressiveness of nymphs reflects

developmental immaturity in both the pheromonal and octopaminergic systems

required for initiating and maintaining aggressive behaviour in adults”.

**Reviewer 2**

**1) Winner and loser effects are usually defined as follows: A previously winning**

**experience increases the winning probability of the next agonistic encounter, whereas a previous losing experience has the opposite effect. Authors did not show in this study that the winners increased their winning probability and the losers decreased it. Authors indicated only increase or decrease of aggression level of fight-experienced crickets. Is there any direct evidences to show correlation between aggression level and agonistic outcomes, since Mustafa wt al. (2019) reported that increase in aggressive level has no advantage of agonistic bouts in zebrafish (Behav Brain Res 270:111942).**

Please note that we also show that winning increased fight duration, and it is known

that animals that persist longer are the ones that win (we have observed this in crickets

many times). Persistence is also a more sensitive measure, particular so for nymphs

where the winners are not always clearly apparent (as mentioned also in the results).

**2) Authors indicated that aggressive behaviour is far less fierce in juveniles and that**

**their interactions do not result in winner or losers effects typical for adults. I agree**

**authors' conclusion according to the results presented in this paper. However, I am**

**very afraid that a rather short period of isolation (48 h) leads to these results. Simmons (1987; ref 7) and Abe et al. (2018; ref 17) have reported that juvenile male crickets exhibit distinguish aggressive behaviours and form discrete dominant-subordinate relationship. In their experiments, juvenile animals are isolated much longer period after final moult of juveniles. In actual, aggressive level of losing crickets increased to 5 after long-term isolation of more than 14 days in this paper (Fig. 6D) that is higher than that of naive adult males (median 4) isolated 48 h (Fig. 3A). Thus, the results might be changed if juveniles isolated more longer are used in this study. Please argue this possibility to Discussion section.**

To accommodate this critic, we now state in the discussion: “Since social isolation is

generally associated with increased aggression, it might be argued that nymphs could

become more aggressive if they were isolated for longer than 48 h as in our study.

However, in adult crickets at least, there is no difference in aggression between

individuals isolated for 1, 2 or 6 days [15]. Furthermore, low aggression in grouped and

briefly (< 1 h) isolated adult crickets has been shown to result from prior social

subjugation, and that isolation merely allows recovery from the loser-effect to a default

aggressive states [15]. Since nymphal, agonistic interactions appear to have no

consequence for their future agonstic behaviour (Fig 3B), it appears unlikely that their

aggressiveness would increase with longer social isolation.

Regarding Fig 6D, the observation that LTI show higher aggression than short term

isolates (STI Fig 3A) is something reported in an earlier paper, and is not surprising

since LTIs have had practically no previous adverse social agonistic experience as

adults (cited from reference 17).

Regarding isolation period, the earlier authors write “Sixth instar larvae (were) placed

individually into small Petri dishes” (ref. 7) or that crickets were isolated “after juveniles

moulted to the final instar stage” (ref. 20), but we found no clear statement when

exactly their aggression was evaluated, but this is now beside the point we think.

**3) As my understanding, two different statistical analyses were performed in Figure 1 and also Figure 2. First analyses is that the occurrence frequency of a particular**

**behavioural act (e.g. antennal fencing) to the same opponent was compared between**

**juvenile and adult groups. And the second analyses is that the difference of juvenile (or adult) responses to 4 opponent groups were compared. Argues of this difference of comparison of groups are rather ambiguous to understand. Authors must clarify this point. If authors want to compare the difference of adult male's response to 4 different opponents simultaneously, significance level alpha must be set 0.05/(3+2+1) = 0.0083. I could not understand meaning of L234-237. I think summarized table(s) of statistical analysis would be helpful to understand the treatments (or groups) of multiple comparisons.**

Sorry, it seems that our original text to the experiments in Figs 1 and 2 have misled the

referee to assume this data is from the same experiment (or is a repeated experiment)

using the same animals, which is not the case. To avoid this confusion, we now give

the methods for these experiments in 2 separate sections, and have changed the

section heading leading to Fig 1 in the text to: “Agonistic responses of nymphs and

adults to each other’s presence” and the text section heading leading to Fig 2 to

“Agonistic responses of nymphs and adults to manual antennal stimulation”.

To clarify that the data in Fig 2 (and all other Figs) are based on fresh observations of

different individuals, we now state in Methods: “Our analysis is based on observations

of 1032 crickets, of which none were used more than once for an experiment or again

in other experiments unless explicitly stated otherwise.”

To avoid any misunderstandings regarding multiple comparisons, we now provide the

following text in Methods (Statistical analysis): “For single comparisons, the

significance level alpha was set to p < 0.05. To compensate for errors due to multiple

comparisons, we applied the Bonferroni correction to alpha for each time the same, or

a mathematically related, set of numerical data was tested more than once. In the

current study, there was never more than two comparisons (alpha is noted in text or

legends).

We would also like to point out, that here is no consensus on when data sets should be

included as part of an entire test family when adjusting test statistics for multiple

comparisons (for arguments and references see [19]). Our standpoint, now given in

the methods, has been accepted e.g. by the journal Animal Behaviour (Elsevier), which

is particularly strict regarding multiple comparisons.

**Minor points**

L44: Delete However - DONE

L184: when adult means when they became adult? Yes, Rephrased

L203-204: meaning is unclear. DONE REWORDED

L249-250 and many places: significance level alpha must be set 0.0083. NO WE

COMPARE 2 GROUPS (SEE POINT 3 ABOVE)

L276: meaning "due to two comparisons" is unclear. Authors compared 4 groups. NO

WE COMPARE 2 GROUPS (SEE POINT 3 ABOVE)

L287-288: delete with alpha set to 0.025 TEXT NOW CHANGE (SEE POINT 3

ABOVE)

L289: against who? IN RESPONSE TO STIMULATION WITH A HEXANE-WASHED

ANTENNA

L421: Contrasting these? NO CHANGE (THIS IS CORRECT HERE WE THINK)

L424: alpha set to 0.017 (= 0.05/3) NO WE COMPARE 2 GROUPS (SEE POINT 3

ABOVE)

**Reviewer 3**

**1) The text reads a bit wordy and repetitive at times and should be streamlined. In a**

**similar way the discussion is a bit repetitive, i.e. results are repeated in detail instead of summarised. Also some key words like “personality” are rather thrown into the text in brackets. I think the MS would gain by briefly/critically explaining these key words a bit more, without over emphasizing.**

We thank this reviewer for offering numerous very helpful suggestions for improvement

in the marked PDF. We have adopted all the changed indicated. We have also

shortened the indicated sections in the discussion and deleted repetitions.

Regarding the term personality: We suspect that this reviewer may find this term

inappropriate, as we do ourselves, but we would prefer to keep back any critical

comments at present. However, to comply with the request, we now use the term

animal personality without quotes and change an introductory sentence to read:

Experiencing aggression, particularly social subjugation (social defeat stress), is

recognised as a major factor inducing depression and depression-like symptoms in

humans and animals [1]. Accumulating evidence also points to links between

aggression and consistent inter-individual behavioural differences, described in recent

years in many vertebrates and invertebrates, and currently referred to as animal

personality [2].

**2) Regarding the figures: Some of the distributions in Figs 3 to 5 look identical, which**

**comes as a surprise, please comment. May be overlaying the original data points with**

**the bar charts could make the data representation more clear.**

Our account was not clear here, and has been changed. For example, the grey bars in

Fig 3 have been changed to a single bar, it depicts the data at the first fight between

“future” winners and losers (light red/light blue bar) - hence the scores are the same.

We only separated the group so that we could make a paired comparison to the same

animals at the second fight – which we still do, but show only one bar. We hope the

new legend makes this clear.

Some of bars in figs 4 and 5 also look similar, particularly for losers. Here the data are

from different animals sets - their performances are however very consistent. We have

tried putting all data points on the graph, but this is detrimental to clarity in the graphs.

**Reviewer 4**

**1) The authors should provide references for their statement of “widely reported”**

**agonistic behavior of pre-adult invertebrates.**

A very good point! We have now changed the text to read: “There are comparatively

few studies of pre-adult aggression in invertebrates [7-9]”, and we know of only one

study in crayfish that investigates its consequence for future behaviour when adult

[10].” Please note: references 8-10 are new.

**2) The direct quotes taken from reference #7 in the introduction (lines 65-67) should be removed; instead, this information should be summarized in the authors’ own words.**

We plea to leave the quotes, it is our explicit intent to cite the author exactly, as we do

not wish to give out interpretation of the author’s statements.

**3) In methods, a clearer explanation of how pauses in activity were ‘deducted’ (line**

**141) is needed.**

DONE. We now write: “On the few occasions that the animals briefly lost contact to

each other during a contest, the stopwatch was paused, so that only the duration of

their interactions was recorded.”

**4) The units of ‘20μl/1 mM CDA’ used by the authors should instead be ‘20 μl of a 1**

**mM CDA’**

DONE - changed as requested

**5) The arrows in Fig 1 should be explained in the legend.** DONE

**6) The authors data show that nymphs do not open their mandibles as wide as adults**

**during antennal stimulation (Fig. 2B), but the opposite is stated in the text (line 304).**

DONE - text amended

**7) ‘Proactive’ (ex. line 426) should be replaced with the word ‘active’ throughout the**

**manuscript.**

DONE

**8) It would have been interesting to see the adult data for male nymphs raised with**

**adult females. This data could have further supported the idea that it was specifically**

**the aggressive attacks by the adult males that were key to these findings. Alternatively, it could have answered the question of whether simply interacting with adults of any sex could influence a nymph’s adult aggressiveness. However, the results do clearly support the importance of a nymph’s social history can impact its future behavior.**

In retrospect, yes this would have been a good addition. But we would not expect any

effect since the female do no intimidate the nymphs (see Fig 6B).
